# Attitudes, motivators, and barriers toward influenza vaccination for children: a study from a conflict-ridden country

**DOI:** 10.1186/s13031-024-00590-9

**Published:** 2024-04-03

**Authors:** Wesam S. Ahmed, Abdulsalam M. Halboup, Arwa Alshargabi, Ahmed Al-mohamadi, Yousf K. Al-Ashbat, Sayida Al-Jamei

**Affiliations:** 1 College of Health and Life Sciences, Hamad Bin Khalifa University, Qatar Foundation, Doha, Qatar; 2 Department of Clinical Pharmacy and Pharmacy Practice, Faculty of Pharmacy, University of Science and Technology, Sana’a, Yemen; 3 Department of Clinical Pharmacy, School of Pharmaceutical Sciences, University Sains Malaysia, Penang, Malaysia; 4 Pharmacy Department, Faculty of Medical Sciences, Saba University, Sana’a, Yemen; 5 Department of Clinical Pharmacy and Therapeutics, Faculty of Pharmacy, Al-Razi University, Sana’a, Yemen

**Keywords:** Seasonal influenza, Influenza vaccine, Children vaccination, Attitudes, Motivators, Barriers, Yemen

## Abstract

**Background:**

Despite the increased recommendations for influenza vaccination, particularly among high-risk groups such as young children, Yemen lacks an influenza vaccination program, and the influenza vaccine is not included in the national immunization regime. This is exacerbated by the country’s fragile infrastructure, as well as the devastating consequences of the ongoing conflict, which include child undernutrition and strained healthcare resources. Thus, the objective of the current study is to assess the public attitudes and perceptions toward vaccinating children against influenza in Yemen.

**Methods:**

A cross-sectional study was conducted by distributing a validated survey questionnaire to potential participants using convenience sampling. Descriptive statistics were used to summarize sociodemographic data, knowledge of influenza vaccines, and attitudes and perceptions regarding vaccinating children against influenza. Logistic regression analysis was employed to identify associations between independent variables and the acceptance of vaccines for children.

**Results:**

A total of 853 eligible individuals, parents and non-parents, successfully completed the survey. The uptake of the influenza vaccine among the participants was notably low as the majority (69.2%) had not previously received the vaccine, although the majority expressed a willingness to get vaccinated in the future (59.4%). The majority (68.5%) were willing to vaccinate children. The largest percentage of the participants who expressed hesitancy toward children’s vaccination cited multiple reasons to reject the vaccine (39.7%), with concerns regarding the safety of the vaccine being the predominant barrier to its acceptance for children (29.6%). On the other hand, motivating factors for vaccinating children included the validation of the vaccine’s safety and efficacy, endorsement of the vaccine by the government and physicians, integration of the vaccine into the national immunization program, and the provision of the vaccine free of charge and through schools. Significant predictors for vaccine acceptance in children included male gender, knowledge of the protective effect of the influenza vaccine, previous receipt of the vaccine, and a willingness to receive the vaccine in the future.

**Conclusions:**

The study highlights the need for educational health campaigns to raise awareness and remove misconceptions regarding influenza and the role, benefits, and availability of its vaccine. These findings can serve as a robust foundation for the future design and implementation of an influenza vaccination program for children in Yemen.

## Background

Seasonal influenza is defined as an acute respiratory tract infection that is caused by the influenza virus [1]. Annually, the disease is responsible for the morbidity of more than one billion individuals globally, resulting in three to five million severe cases and between 290 and 650 thousand influenza-related mortality [2]. The most common symptoms caused by the uncomplicated infection include fever, cough, fatigue, headache, sore throat, muscle pain, and runny nose [3, 4]. Moreover, complications of influenza are also common and include sinus and ear infections, pneumonia, and worsening of co-morbid conditions such as heart failure, asthma, and diabetes [5, 6]. High-risk categories of influenza complications include pregnant females, immunocompromised patients, adults of more than 65 years of age, individuals with chronic conditions, and children younger than five years of age, with those younger than two years old being at even increased risk of influenza-related complications and hospitalization [7–9].

The seasonal influenza vaccine serves as a key preventive measure against influenza and its associated complications, offering numerous benefits, such as protection against influenza infection, hospitalization, and disease severity [10–18]. The Centers for Disease Control and Prevention recommends that all individuals aged 6 months or older receive the vaccine annually, with a higher priority assigned to high-risk groups and healthcare workers [19–23].

Yemen, a low-income Middle Eastern developing country, has been experiencing an ongoing armed conflict since 2015. In 2010, the country initiated a surveillance program for severe acute respiratory infections [24]. However, due to the outbreak of war and restricted access to healthcare facilities, the electronic disease early warning system (eDEWS) was strengthened as an integrated system for infectious diseases in the country, beginning in 2016 [25, 26]. Reportedly, there was a two-fold increase in influenza incidence during the 2018–2019 season, with a 22% case fatality rate [24, 27]. According to a recently published study, influenza infection in children under 15 years old constituted approximately 19% of all influenza cases in Yemen. The same study identified age under five as a risk factor for influenza and influenza-associated hospitalization in the country [27]. This is alarming as the country’s healthcare system has collapsed because of the ongoing conflict, and the increased influenza cases have overwhelmed hospitals and intensive care units, depleting already scarce healthcare resources. In addition to COVID-19, the country faces an exacerbated influenza burden due to the circulation of other fever-causing, neglected tropical infections, including malaria, dengue fever, and chikungunya fever [28]. Despite the huge burden created by seasonal influenza, the seasonal influenza vaccine is not implemented in the national immunization program, and the country has no seasonal influenza vaccination policy applied to the public or subgroups [29]. Moreover, no prior surveillance programs or awareness campaigns that target influenza and the importance of its vaccine in children have been applied in the country. Additional challenges in Yemen, such as political conflict, personal safety, food security, and weak infrastructure, stand as potential barriers to vaccination [28, 30].

Many countries implement specialized seasonal influenza vaccination programs for children. For instance, the US Centers for Disease Control and Prevention recommend influenza vaccination for all children aged 6–59 months and for children aged 5–17 years with high-risk conditions [31]. Achieving high vaccination rates among children is of paramount importance, as it does not only provide protection to the vaccinated children but also extends this protection to their families and the broader community. Additionally, immunizing schoolchildren can yield further benefits for educational institutions, including reductions in both student and teacher absenteeism, thereby enhancing the capacity of schools to fulfill their primary educational mission [32]. However, in Yemen, these vaccination recommendations are not currently being implemented [28, 29]. Therefore, to effectively extrapolate these recommendations, a responsible approach should be taken, drawing upon the current situation of the country and the public’s knowledge of influenza, as well as their attitudes toward its vaccine. Hence, the objective of the present study is to assess the public’s attitudes, perceived motivators, and barriers regarding the administration of the seasonal influenza vaccine to children. This study aims to provide valuable insights for the future, evidence-based design and implementation of vaccination programs against seasonal influenza for children in Yemen, with the overarching goal of improving overall vaccination coverage and its associated population-wide health benefits.

## Methods

### Study design and participants

The study employed a two-phase sampling approach. The initial phase involved face-to-face survey distribution and was conducted from March 2019 to February 2020 but was subsequently halted due to the COVID-19 outbreak. During this phase, the survey was administered by experienced interviewers to eligible participants at public locations in Sana’a city such as markets, parks, and universities. In the second phase, an online survey distribution method was employed, which took place between July and October 2020. During this phase, the survey was disseminated to members of the public through social media platforms (WhatsApp and Facebook), attracting participants from different cities across the country. Eligible participants included those who were at least 18 years of age, autonomous, and able to read and comprehend the Arabic language. The informed consent was obtained orally during the in-person recruitment phase. For the online sampling, the consent statement was integrated into the online questionnaire and in the participation invite that was sent through social media platforms. The sample size was calculated using the Daniel sample size formula [33], assuming a binomial distribution, a confidence interval of 95%, a margin of error of 5%, and a distribution rate of 50%, commonly used for conservative estimations when the true proportion is unknown. The minimum sample size was determined to be 377.

### Survey questionnaire

The questionnaire had been previously validated [34] and was distributed in the Arabic language. The survey incorporated various response formats, including “Yes,” “No,” and “I am not sure,” as well as multiple-choice questions, multiple-checkbox items, and Likert scale items. The survey was structured into four sections. The first section aimed to collect demographic information from the participants including age, gender, location, education level. Additionally, the first section assessed the pregnancy status, whether participants were from the medical field, possessed health insurance coverage, or had any chronic medical conditions. The second section was designed to evaluate participants’ knowledge, attitudes, and practice related to the influenza vaccine. The third section solicited public’s attitudes toward vaccinating children and included follow-up, multiple checkboxes, questions assessing their perceived barriers to the vaccination of children. The final section of the questionnaire sought to identify the motivators that will influence participants to vaccinate children.

### Data analysis

The data were analyzed using IBM SPSS Statistics version 27.0 for Windows® (IBM Corp., Armonk, NY, USA). Categorical variables, including participants’ demographic data and attitudes toward influenza vaccination, were presented as frequencies and percentages. Regarding the motivation of the participants toward vaccinating children, the five-point Likert scale of the six questions was transformed into a three-point Likert scale, ranging from “strongly agree/agree” to “strongly disagree/disagree,” with scores assigned from 1 to 3. The final scores ranged from 6 to 18, providing a measurement of participants’ motivation levels for future vaccination decisions, with lower scores indicating stronger motivation. The first quartile (25th percentile) was used as a cutoff point to divide participants’ motivation into two subcategories: “positive” and “negative” motivation. Subsequently, univariate logistic regression was employed to examine the relationship between participants’ willingness to vaccinate children in the future (dependent variable) and other independent variables including sociodemographic data, awareness to seasonal influenza and its vaccine, and motivation to vaccinate children. Variables with a p-value < 0.25 in the univariate logistic regression were included in the multivariable logistic regression model to construct a predictive model for assessing participants’ willingness to vaccinate children in the future. Odds ratios were calculated to quantify the impact of each predictor on participants’ willingness. A confidence interval of 95% was utilized in the analysis. A p-value of less than 0.05 was considered statistically significant.

## Results

The sociodemographic characteristics of the participants are reported in Table 1. A total of 853 participants completed the questionnaire. The response rate (on-site only) and the completion rate (on-site & online) were 55% and 68% respectively. The median age of the participants was 29 years old (IQR = 11). There were about twice as many male participants (65%, n = 558) than females. Most participants were enrolled in or had a higher education degree either undergraduate (70%, n = 599) or postgraduate (13%, n = 112). The majority were from large governorates such as Sana’a (34%, n = 293), Taiz (23%, n = 192), and Ibb (11%, n = 96). In addition, most participants were non-smokers (87%, n = 745), with no known chronic medical conditions (86%, n = 732), not covered by medical insurance (73%, n = 621), and not enrolled in the medical field (61%, n = 517).

**Table 1:** Sociodemographic and other characteristics of the participants (n = 853)

Characteristic		n	(%)
Age in years, Median (IQR): 29 (11)			
Gender			
	Male	558	(65.4)
	Female	295	(34.6)
Education level			
	Secondary school	142	(16.6)
	Undergraduate	599	(70.2)
	Postgraduate	112	(13.1)
Governorate			
	Sana’a	293	(34.3)
	Taiz	192	(22.5)
	Ibb	96	(11.3)
	Hodidah	47	(5.5)
	Hajah	33	(3.9)
	Others	192	(22.5)
Are you enrolled in the medical field?			
	Yes	336	(39.4)
	No	517	(60.6)
Do you have any chronic medical conditions?			
	Yes	120	(14.1)
	No	732	(85.9)
Are you covered by health insurance?			
	Yes	232	(27.2)
	No	621	(72.8)
Are you a smoker?			
	Yes	108	(12.7)
	No	745	(87.3)
Are you pregnant?			
	Yes	16	(5.3)
	No	279	(94.7)

### Knowledge, attitudes and practice toward influenza vaccine

The largest percentage of the participants were aware that influenza vaccine protects against influenza-related infections rather than other respiratory tract infections such as COVID (42%, n = 361). However, the largest proportion did not know that the seasonal influenza vaccine shall be taken annually (47%, n = 403). Physicians were the main reported source of information related to influenza and its vaccine (43%, n = 364). Regarding practice toward influenza vaccine, the majority reported never receiving the influenza vaccine (69%, n = 590), although the majority were willing to take the vaccine in the future (59%, n = 507) (Table 2).

**Table 2:** Participants knowledge and attitudes related to influenza vaccine (n = 853)

Item		n	(%)
Your primary source of information about influenza and its vaccine			
	Magazine and newspapers	212	(24.9)
	Doctor	364	(42.8)
	TV	222	(26.1)
	Others	55	(6.1)
Do you know that the seasonal influenza vaccine shall be taken annually?			
	Yes	240	(28.1)
	No	403	(47.2)
	Not sure	210	(24.6)
Do you know that influenza vaccine can protect against swine flu but not COVID?			
	Yes	361	(42.3)
	No	249	(29.2)
	Not sure	243	(28.5)
Have you ever received the seasonal influenza vaccine?			
	Yes	113	(13.2)
	No	590	(69.2)
	Not sure	150	(17.6)
Are you willing to receive the vaccine in the future			
	Yes	507	(59.4)
	No	146	(17.1)
	Not sure	200	(23.4)

### Attitudes, barriers, and motivators to seasonal influenza vaccination in children

Almost half of the participants had children aged less than 5 years old (47%, n = 397). Most of those were between 3 and 5 years of age (64%, n = 254). The majority of the respondents were willing to give the vaccine to children (69%, n = 584). Most of those who were reluctant to giving the vaccine to children had more than one reason to reject the vaccine (40%, n = 106), and cited concerns regarding the vaccine’s safety as a main reason for rejecting children vaccination (30%, n = 79) (Table 3). On the other hand, most participants were motivated to give the vaccine to children if it was advocated by the government (73%, n = 599), recommended by the physician (76%, n = 621), included in the national immunization regime (78%, n = 645), validated for safety and efficacy (79%, n = 654), provided by the school (60%, n = 489) and free of charge (69%, n = 566). However, on an individual basis, only half of the participants were positively motivated by these criteria (50%, n = 414) (Table 4).

**Table 3:** Attitudes and barriers to seasonal influenza vaccination in children

Item		n	(%)
Do you have children who are aged < 5 years old?	No	456	(53.5)
	Yes	397	(46.5)
If yes, please specify the age	< 6 months	36	(9.1)
	6 months to 2 years	107	(27.0)
	3–5 years	254	(64.0)
Would you agree to vaccinate your children against influenza in the future?	Yes	584	(68.5)
	No	153	(17.9)
	Not sure	116	(13.6)
If not, please choose the reason(s) of not willing to vaccinate your children	Doubt regarding the efficacy of the vaccine	21	(7.9)
	Fear of catching influenza	25	(9.4)
	Do not know of the vaccine availability	17	(6.4)
	Doubt regarding the safety of the vaccine	79	(29.6)
	Not considering influenza as a threat	8	(3.0)
	More than one reason	106	(39.7)
	Others	11	(4.1)

**Table 4:** Factors motivating participants to consider vaccinating children against influenza in the future

Item (You would give the vaccine to your children if:)		n	(%)
It is encouraged by the government	Strongly agree/agree	599	(73.0)
	Not sure	138	(16.8)
	Strongly disagree/disagree	84	(10.2)
It is included in the national immunization program	Strongly agree/agree	645	(78.3)
	Not sure	124	(15.0)
	Strongly disagree/disagree	55	(6.7)
It is more validated for safety and efficacy	Strongly agree/agree	654	(79.2)
	Not sure	114	(13.8)
	Strongly disagree/disagree	58	(7.0)
It is provided though school	Strongly agree/agree	489	(60.1)
	Not sure	153	(18.8)
	Strongly disagree/disagree	171	(21.0)
It is recommended by the physician	Strongly agree/agree	621	(76.3)
	Not sure	121	(14.9)
	Strongly disagree/disagree	72	(8.8)
It is offered free of charge	Strongly agree/agree	566	(69.1)
	Not sure	152	(18.6)
	Strongly disagree/disagree	101	(12.3)
Overall motivation	Positive Motivation	414	(49.8)
	Negative Motivation	418	(50.2)

### Predictors for willingness to provide the influenza vaccine to children

The outcomes of the univariate logistic regression, focusing on individual predictors (P-value < 0.25), indicate that the willingness to vaccinate children was significantly associated with male gender of the participants, location, positive motivation, being enrolled in the medical field, previous vaccination, and willingness to get vaccinated in the future. Knowledge about the annual schedule of the vaccine and that it only protects against influenza virus were both associated with positive attitude toward vaccinating children. We subjected these factors to subsequent multivariable logistic regression to investigate factors that exhibited significant and independent association with willingness to vaccinate children (P ≤ 0.05, AOR > 1). Five variables fulfilled the criteria, these were male gender (P < 0.027, AOR = 1.544), previous vaccination (P < 0.003, AOR = 2.995), willingness to receive the vaccine (P < 0.001, AOR = 6.922), positive motivation (P < 0.001, AOR = 4.555), and knowledge of the influenza vaccine protection (P = 0.015, AOR = 1.635) (See Table 5).

**Table 5:** Predictors for willingness to provide the influenza vaccine to children in the future

Variable		Dependent variable: Willingness to give the flu vaccine to your children in the future (0: No/not sure, 1: Yes)			
		Univariate logistic regression		Multivariable logistic regression	
		COR (95% C.I.)	P value	AOR (95% C.I.)	P value
Age	≤ 30 years	Reference			
	> 30 years	0.887 (0.662, 1.188)	0.423		
Gender	Female	Reference		Reference	
	Male	1.797 (1.333, 2.422)	< 0.001^*^	1.544 (1.051, 2.268)	0.027^*^
Education level	Secondary school	Reference			
	Diploma and above	0.860 (0.579, 1.277)	0.455		
Governorate	Sana’a	Reference			
	Others	1.413 (1.047, 1.908)	0.024^*^	1.015 (0.691, 1.493)	0.938
Are you enrolled in the medical field?	No	Reference			
	Yes	1.448 (1.070, 1.958)	0.016^*^	1.235 (0.842, 1.810)	0.281
Do you have any chronic medical conditions?	No	Reference			
	Yes	1.041 (0.686, 1.580)	0.851		
Are you covered by health insurance?	Yes	Reference			
	No	1.023 (0.740, 1.415)	0.890		
Are you pregnant?	Yes	Reference			
	No	0.566 (0.200, 1.606)	0.285		
Do you have children who are aged < 5 years old?	No	Reference			
	Yes	0.901 (0.674, 1.203)	0.478		
Do you know that influenza vaccine shall be taken annually?	No/Not sure	Reference		Reference	
	Yes	2.045 (1.440, 2.904)	< 0.001^*^	1.206 (0.770, 1.887)	0.413
Do you know that influenza vaccine can protect against swine flu but not COVID?	No/Not sure	Reference		Reference	
	Yes	2.302 (1.690, 3.137)	< 0.001^*^	1.635 (1.102, 2.426)	0.015^*^
Have you ever had the seasonal influenza vaccine?	No/ not sure	Reference		Reference	
	Yes	3.718 (2.082, 6.639)	< 0.001^*^	2.995 (1.463, 6.131)	0.003^*^
Are you willing to receive the vaccine in the future	No/ Not sure	Reference		Reference	
	Yes	7.988 (5.755,11.087)	< 0.001^*^	6.922 (4.817, 9.945)	< 0.001^*^
Motivation	Negative Motivation	Reference		Reference	
	Positive Motivation	5.97 (4.254,8.380)	< 0.001^*^	4.555 (3.121, 6.645)	< 0.001^*^

A p-value of < 0.25 in univariate analysis was included in the multivariable analysis. COR: Crude odd ratio; AOR: adjusted odd ration; star sign (*) and bold text indicate significant variables

## Discussion

The prevalence of seasonal influenza in Yemen has shown a notable increase over the past decade, reaching twice the average incidence during the 2018–2019 season [24, 27, 35, 36]. This escalation has placed a heightened burden on the already strained healthcare system, with one-fifth of these cases occurring in children under 15 years of age [27]. The situation is aggravated by the limited availability of healthcare resources in the country, a challenge commonly observed in conflict-ridden regions, which further magnifies the impact of seasonal influenza in the country [28]. Exacerbating these issues is the absence of established seasonal influenza immunization programs as well as awareness campaigns to the availability and value of the seasonal influenza vaccine [29]. This study represents the first large-scale investigation in Yemen, focusing on public attitudes toward influenza vaccination in children and their perceptions of the vaccine. The insights gained from this study are poised to guide the development of nationwide influenza vaccination programs for children, ensuring equitable access to this vital preventive measure.

Assessing participants’ knowledge of the vaccine revealed that the highest percentage of participants were aware that the seasonal influenza vaccine can only provide protection against influenza-related infections. However, this awareness was only observed in less than half of the total sample. It is worth noting that a similar lack of knowledge about influenza has been observed among parents in other studies conducted in the region, such as Saudi Arabia and Jordan [34, 37], and among adults in general [34, 38–41]. In this regard, a large meta-analysis conducted in the United States indicated that the general public tends to possess only basic and rather limited knowledge about influenza-related topics [42]. On the other hand, the largest proportion of participants were unaware that the seasonal influenza vaccine should be taken annually. This underscores the imperative need for educational campaigns to enhance awareness about influenza, as well as to convey information about the vaccine’s role and availability. Physicians emerged as the primary source of information about influenza and its vaccine for participants, which is consistent with findings from previous studies conducted both within the region and globally [34, 37, 43–45]. Therefore, physicians can play a pivotal role in disseminating timely and concise updates regarding seasonal influenza and its vaccine. However, participants practice concerning vaccine uptake was suboptimal, with fewer than 15% reported prior vaccination against influenza. This aligns with findings from previous studies in the region, including Saudi Arabia, Lebanon, Jordan, and Turkey, where low vaccination uptake has also been documented [34, 46–48]. It is noteworthy, though, that most participants expressed a willingness to receive the vaccine in the future. A prior study has indicated that the lack of awareness about vaccine availability poses a significant hurdle to adult vaccination in the country [28]. This once again emphasizes the critical need for active awareness campaigns to promote awareness of the vaccine’s availability [49, 50].

Assessing attitudes toward vaccinating children revealed that the majority of participants expressed a willingness to vaccinate children against seasonal influenza, consistent with prior findings from the region [34, 37, 51]. A substantial portion of participants who were hesitant about vaccinating children cited multiple reasons for their reluctance, with particular emphasis on concerns related to vaccine safety. On the other hand, a significant portion of our participants indicated that their motivation to vaccinate children was influenced by the criteria outlined in Table 4. Notably, there was a high degree of consensus among participants regarding the importance of safety and efficacy testing of the vaccine, underscoring the need to address this concern in future children’s vaccination campaigns. Indeed, the concern regarding the safety and efficacy of the influenza vaccine was a common barrier to vaccination in the region not only for children [34, 37, 51–53] but also for adults [47, 54–56]. However, in one study that included participants from the six Gulf Cooperation Counsel (GCC) countries, concerns regarding the safety and efficacy of the vaccine were reported as the least cited barriers to vaccination [57], probably suggesting a higher trust in the vaccination regimes in these countries. Conversely, the criteria that met with the most resistance in our study was the provision of the vaccine by schools. This observation suggests that there may be lower levels of trust among participants in school-based vaccination protocols compared to other entities, such as governmental health authorities, a factor that should be carefully considered when designing future vaccination strategies for children.

Assessing predictors for positive attitudes toward children’s vaccination revealed that individuals who had previously received the vaccine and those who expressed a willingness to vaccinate themselves were more inclined to consent to vaccinating children, compared to those who did not share these characteristics. These findings align with the results of a previous study conducted in Saudi Arabia [37]. Consequently, enhancing vaccination coverage among parents and optimizing their attitudes toward the vaccine would have a positive impact on the vaccination status of children. Furthermore, a significant association was observed between female gender and influenza vaccine hesitancy, consistent with previous reports from the region [54] and globally [58–61]. Interestingly, in a study from Saudi Arabia, the female gender was associated with stronger positive attitudes toward vaccinating children, indicating potential variations in gender-related attitudes across different contexts. Nonetheless, the exact mechanisms underlying the gender effect on vaccine acceptance remain less explored in the literature [61]. Another positive predictor of vaccine acceptance was knowledge of influenza vaccine protection. This underscores the importance of awareness campaigns aimed at strengthening knowledge and dispelling misconceptions regarding the influenza vaccine, as such efforts have previously led to increased vaccination rates among children, as reported in Jordan [53].

The limitations of the present study align with those commonly associated with cross-sectional study designs, notably including the potential for both selection bias and recall bias. It is important to acknowledge that our sampling approach, although inclusive of participants from various cities in the country, remains a convenience sampling approach by definition. Consequently, the ability to generalize the findings obtained through this approach is inherently limited when compared to random sampling, albeit the latter would pose considerable challenges given the current situation in the country. In spite of these limitations, the findings derived from our study hold substantial value for the design of vaccination programs targeting children in the country, and they would serve as a critical stepping stone for future population-based studies addressing other high-risk groups of influenza infection with the ultimate goal of augmenting influenza vaccination coverage within the country.

## Conclusion

The current study shows a low vaccination uptake among study participants with consensus to self-receive the vaccine and provide it to children in the future. There was a gap of knowledge with regards to the vaccine protection and its annual schedule. Our study recognized major barriers and motivators to willingness to vaccinate children and identified concerns regarding the safety and efficacy as important factors that need to be addressed and considered in the future design and implementation of vaccination programs for children. Moreover, the study identified predictors for vaccine acceptance in children and highlighted knowledge of influenza vaccine protection and positive vaccination status as major determinants of this acceptance. The present study provides valuable insights for the implementation of educational campaigns and influenza vaccination regimes for children in the country. Additionally, the study will aid in the design of future population-based studies targeting other high-risk groups susceptible to influenza complications that will ultimately inform the design and implementation of a rational national immunization policy against seasonal influenza within the country.

## Data Availability

The datasets used and/or analysed during the current study are available from. the corresponding author on reasonable request.
